# Supplementary material for: Machine learning based tuberculosis (ML-TB) health predictor model: early TB health disease prediction with ML models for prevention in developing countries
Source: PeerJ Comput Sci. 2024 Oct 16;10:e2397. doi: 10.7717/peerj-cs.2397 (PMC11623013; doi:10.7717/peerj-cs.2397)
Supplement: Supplemental Information 4 [file peerj-cs-10-2397-s004.docx]

**University of Sindh Jamshoro**
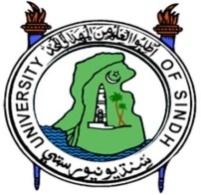


**Institute of Mathematics and Computer Science (IMCS)**

**M.Phil. (Research Project)**

**TB SCREENING QUESTIONNAIRE**

Date: ________________

**Personal Information**

Name:__________________Age:________________ Gender:___________________

Weight:_________________Height:______________Occupation:________________

Religion:________________Nationality:___________D.O.B:____________________

Address:______________________________________________________________

**Investigation**

**1. Have you experienced any of the following signs and symptoms in the recent past?**

a. Productive cough for more than 3 weeks ☐Yes ☐No

b. Hemoptysis (coughing up blood) ☐Yes ☐No

c. Sputum with blood ☐Yes ☐No

d. Evening rise temperature ☐Yes ☐No

e. Unexplained weight loss ☐Yes ☐No

f. Poor Appetite ☐Yes ☐No

g. Weakness/Fatigue ☐Yes ☐No

h. Chills or night sweats for no known reason ☐Yes ☐No

i. Persistent shortness of breath ☐Yes ☐No

j. Dyspnea on exertion ☐Yes ☐No

k. Chest Pain ☐Yes ☐No

l. Abnormal sound on respiration ☐Yes ☐No

m. Pulmonary Effusion ☐Yes ☐No

**2. Have you suffered any of the following risk factor in the recent past time?**

a. HIV/AIDS ☐Yes ☐No

b. Smoking ☐Yes ☐No

c. Crowding ☐Yes ☐No

d. Malnutrition ☐Yes ☐No

e. Chronic Lung Disease ☐Yes ☐No

f. Silicosis ☐Yes ☐No

g. Diabetes Mellitus ☐Yes ☐No

**3.** **Have you had contact with anyone with active TB disease in the recent past?** ☐Yes ☐No

**4**. **Do you have a medical condition, or are you taking medications, which suppress your immune system?**  ☐Yes ☐No

**5. Is your ESR value in blood CP reports exceeds 100?** ☐Yes ☐No

**6. Have you been a resident and/or employee of high-risk congregate settings (e.g., correctional facilities, long-term care facilities, and homeless shelters)?** ☐Yes ☐No

**7. Have you been a volunteer or health-care worker who served clients who are at increased risk for active TB disease?**  ☐Yes ☐No

**8.** **Have you ever been a member of any of the following groups that may have an increased incidence of latent M. tuberculosis infection or active TB disease – medically underserved, low-income, or abusing drugs or alcohol?**  ☐Yes ☐No

**9. Have you ever been tested for TB?** ☐Yes ☐No

**If Yes, Please mention test name: ______________ Dated: _____________**

**10. Was your test positive for TB?** ☐Yes ☐No

**11.** **Have you ever been prescribed the treatment for TB disease?** ☐Yes ☐No

**If yes, what medications were you given?**

**_______________________________________________________________**

**12. Have you completed your treatment?** ☐Yes ☐No

**13. Have you been cured from TB disease completely?** ☐Yes ☐No

**Any Other Information:**

**Comments/Feedback:**

________________________________________________________________________________________________________________________________________________

**Thank You**
